# Sustaining a Young Self-Perception: Feeling Younger or Redefining Age Boundaries?

**DOI:** 10.1093/geroni/igaf122.220

**Published:** 2025-12-31

**Authors:** M Clara de Paula Couto, Fiona Rupprecht, Klaus Rothermund, Jana Nikitin

**Affiliations:** Friedrich-Schiller University Jena, Jena, Thuringen, Germany; University of Vienna, Vienna, Wien, Austria; Friedrich-Schiller University Jena, Jena, Thuringen, Germany; University of Vienna, Vienna, Wien, Austria

## Abstract

Our study investigates how individuals counteract societal negative attitudes towards aging by striving to maintain a youthful self-image, either by feeling younger than their actual age or by redefining what they consider “old age”. Based on the dual-process theory of developmental regulation, feeling younger is the result of an assimilative strategy, while adjusting the concept of old age is an accommodative strategy. We examined these strategies across different life domains, such as family, friendships, and work, hypothesizing that people emphasize youth-preserving strategies in areas critical to their identity. We focused on whether older adults more commonly adjust their definition of old age in important life domains (accommodative response), versus younger adults preferring to feel younger in domains that are deemed important (assimilative response). Analyzing data from 768 participants aged between 30 to 80 years through multilevel analysis, our research confirmed these hypotheses. Findings reveal that individuals indeed work to maintain a youthful identity in domains they find important. Younger participants felt younger in crucial life domains, whereas older participants adjusted their old age threshold in personally relevant domains. This behavior suggests a societal tendency to value youth, with individuals using both old age perception adjustment and feelings of youthfulness to protect their self-image and avoid the stigma of being old.

